# Benchmarking Outcomes after Ablative Radiotherapy for Molecularly Characterized Intrahepatic Cholangiocarcinoma

**DOI:** 10.3390/jpm11121270

**Published:** 2021-12-01

**Authors:** Brian De, Ibrahim Abu-Gheida, Aashini Patel, Sylvia S. W. Ng, Mohamed Zaid, Connor P. Thunshelle, Dalia Elganainy, Kelsey L. Corrigan, Michael K. Rooney, Milind Javle, Kanwal Raghav, Sunyoung S. Lee, Jean-Nicolas Vauthey, Ching-Wei D. Tzeng, Hop S. Tran Cao, Ethan B. Ludmir, Bruce D. Minsky, Grace L. Smith, Emma B. Holliday, Cullen M. Taniguchi, Albert C. Koong, Prajnan Das, Eugene J. Koay

**Affiliations:** 1 Department of Radiation Oncology, The University of Texas MD Anderson Cancer Center, Houston, TX 77030, USA; ibrahimabugheida@gmail.com (I.A.-G.); aashini.r.patel@uth.tmc.edu (A.P.); sylvia.ng@sunnybrook.ca (S.S.W.N.); mmzaid@mdanderson.org (M.Z.); cthunshelle@gmail.com (C.P.T.); dalia_elganainy@dfci.harvard.edu (D.E.); klcorrigan@mdanderson.org (K.L.C.); mkrooney@mdanderson.org (M.K.R.); ebludmir@mdanderson.org (E.B.L.); bminsky@mdanderson.org (B.D.M.); glsmith@mdanderson.org (G.L.S.); ebholliday@mdanderson.org (E.B.H.); ctaniguchi@mdanderson.org (C.M.T.); ackoong@mdanderson.org (A.C.K.); prajdas@mdanderson.org (P.D.); 2 Department of Gastrointestinal Medical Oncology, The University of Texas MD Anderson Cancer Center, Houston, TX 77030, USA; mjavle@mdanderson.org (M.J.); kpraghav@mdanderson.org (K.R.); sslee1@mdanderson.org (S.S.L.); 3 Department of Surgical Oncology, The University of Texas MD Anderson Cancer Center, Houston, TX 77030, USA; jvauthey@mdanderson.org (J.-N.V.); cdtzeng@mdanderson.org (C.-W.D.T.); hstran@mdanderson.org (H.S.T.C.)

**Keywords:** cholangiocarcinoma, mutation, genetic, genomic, radiotherapy

## Abstract

We have previously shown that ablative radiotherapy (A-RT) with a biologically effective dose (BED_10_) ≥ 80.5 Gy for patients with unresectable intrahepatic cholangiocarcinoma (ICC) is associated with longer survival. Despite recent large-scale sequencing efforts in ICC, outcomes following RT based on genetic alterations have not been described. We reviewed records of 156 consecutive patients treated with A-RT for unresectable ICC from 2008 to 2020. For 114 patients (73%), next-generation sequencing provided molecular profiles. The overall survival (OS), local control (LC), and distant metastasis-free survival (DMFS) were estimated using the Kaplan–Meier method. Univariate and multivariable Cox analyses were used to determine the associations with the outcomes. The median tumor size was 7.3 (range: 2.2–18.2) cm. The portal vein thrombus (PVT) was present in 10%. The RT median BED_10_ was 98 Gy (range: 81–144 Gy). The median (95% confidence interval) follow-up was 58 (42–104) months from diagnosis and 39 (33–74) months from RT. The median OS was 32 (29–35) months after diagnosis and 20 (16–24) months after RT. The one-year OS, LC, and intrahepatic DMFS were 73% (65–80%), 81% (73–87%), and 34% (26–42%). The most common mutations were in *IDH1* (25%), *TP53* (22%), *ARID1A* (19%), and *FGFR2* (13%). Upon multivariable analysis, the factors associated with death included worse performance status, larger tumor, metastatic disease, higher CA 19-9, PVT, satellitosis, and *IDH1* and *PIK3CA* mutations. *TP53* mutation was associated with local failure. Further investigation into the prognostic value of individual mutations and combinations thereof is warranted.

## 1. Introduction

Intrahepatic cholangiocarcinoma (ICC) is the second most common primary liver malignancy, with rates of incidence and mortality increasing 4% each year [1,2,3]. While surgery is the only known potentially curative treatment for ICC, only 12% of patients have localized disease upon presentation and less than 40% of these patients ultimately undergo resection [4,5]. For patients with unresectable disease, cytotoxic chemotherapy has traditionally been used for treatment; however, it has short-lived efficacy owing to the rapid development of resistance [6]. The prognosis of unresectable ICC is poor, with a reported median overall survival (OS) of 3 to 12 months [7,8,9]. Approximately 70% of the patients with ICC die of tumor-related liver failure (TRLF) associated with the inadequate control of the primary or satellite lesions, which destroy or obstruct the neighboring parenchyma, vasculature, or bile ducts [10,11].

Recent investigation in unresectable ICC has centered on the use of local therapies and the development of molecularly targeted systemic therapies. Retrospective data from our institution have shown that ablative radiotherapy (A-RT) with biologically effective doses (BED) ≥ 80.5 Gray (Gy) for unresectable ICC was associated with improved 3-year rates of local control (LC; 73% versus 38%) and overall survival (OS; 73% versus 38%), likely owing to lower rates of TRLF [11,12,13]. In parallel, studies characterizing the mutational landscape of ICC have led to the development of drugs selectively targeting isocitrate dehydrogenase 1 (*IDH1*) and fibroblast growth receptor 2 (*FGFR2*) [14,15,16]. Nevertheless, the prognostic significance of the larger mutational landscape remains poorly understood because of the rarity of the disease [17,18].

Despite the parallel efforts of large-scale sequencing and investigation into the use of ablative dose RT for the treatment of ICC, the outcomes of ICC patients treated with ablative dose RT based on genetic alterations have not been described. This study aims to explore the differential outcomes stratified by mutational status and to provide benchmarks for future investigations.

## 2. Materials and Methods

### 2.1. Patient Selection, Workup, and Treatment

After approval by the Institutional Review Board (PA14-0646), we identified 156 consecutive patients aged ≥ 18 years with biopsy-confirmed ICC treated with A-RT between 2008 and 2020 at MD Anderson Cancer Center. All patients received standard pre-treatment evaluation, including history and physical examination, blood work including liver function tests and tumor markers, internal review of pathology slides by dedicated hepatobiliary pathologists, and imaging including computed tomography of the chest/abdomen/pelvis, and possible liver magnetic resonance imaging at the discretion of the treating physician. Radiation therapy simulation and treatment details have been previously described [13,19]. Patients were assessed 4–6 months following completion of RT and every 3–6 months thereafter. All post-treatment scans were reviewed by a hepatobiliary radiologist and by the treating radiation oncologist.

### 2.2. Data Collection

We abstracted patient demographic, disease, and treatment details from patient medical records. Any disease recurrence within the RT field was recorded as local progression. Any liver disease progression outside the RT field was recorded as an intrahepatic distant metastasis, while any new nodal or extrahepatic disease was coded as extrahepatic metastasis.

### 2.3. Mutational Profiling

The majority of molecularly characterized patients were sequenced using biopsy tissue at initial diagnosis, prior to the start of any therapy. A hybrid capture-based next-generation sequencing assay was utilized for patient samples in a Clinical Laboratory Improvement Amendments (CLIA)-certified laboratory. Mutations were characterized using either solid tumor tissue or circulating cell-free DNA (cfDNA) to screen for single nucleotide variants, insertions/deletions, copy number gains, and gene fusions. Tissue-based testing assessed up to 324 genes and associated biomarkers, with FoundationOne CDx (Foundation Medicine, Cambridge, MA, USA) serving as the most recently utilized assay [20]. Liquid biopsies were performed by obtaining peripheral blood, isolating plasma, and extracting DNA after which genomic profiling was performed. Most commonly, liquid biopsy genomic profiling was obtained using the FoundationACT, FoundationOne Liquid, or FoundationOne Liquid CDx (Foundation Medicine, Cambridge, MA, USA) assays [21,22]. Additionally, immunohistochemistry was used for DNA mismatch repair enzymes (*MLH1*, *MSH2*, *MSH6*, *PMS2*), and further testing was ordered at the discretion of the treating medical oncologist.

### 2.4. Statistical Analysis

Baseline characteristics were summarized using descriptive statistics. The median follow-up time with associated confidence interval was calculated using the reverse Kaplan–Meier method. OS was defined as the latency from the start of RT to death of any cause or last follow up. We defined composite endpoints local failure-free survival (LFFS), intrahepatic distant metastasis-free survival (DMFS), and extrahepatic DMFS as the latencies between the start of RT and recurrence, death, or last follow up. Time-to-event endpoints were analyzed using the Kaplan–Meier method and compared using the log-rank test. Univariate and multivariable Cox analyses were used to determine associations with outcomes. The proportional hazards assumptions for all univariate and multivariable models were evaluated using χ2 tests of Schoenfeld residuals. Tests of the proportional hazards assumptions for death, local progression or death, intrahepatic distant progression or death, and extrahepatic distant progression or death using Schoenfeld residuals all yielded *P* > 0.05 and, thus, we failed to reject the null hypotheses that the hazards were proportional. We used a *P*-value threshold of ≤0.05 on univariate analysis for overall survival and ≤0.10 on univariate analysis for local failure to select variables for inclusion in each corresponding multivariable model; thresholds were deliberately chosen to limit the number of variables in final models to ≤ 10 in order to mitigate the risk of multivariable model overfitting. Statistical analysis was performed with Stata Version 16.1 (StataCorp, College Station, TX, USA).

## 3. Results

### 3.1. Baseline Characteristics

The patient, disease, and treatment characteristics are shown in Table 1. The majority of the patients were female (54%) and had Eastern Cooperative Oncology Group (ECOG) performance status of 0–1 (93%). The median age at the start of the A-RT was 66 years (range: 31–89). Satellitosis was detected in 51% of patients. The median dominant tumor size was 7.3 (range: 2.2–18.2) cm. The summary stage was I-III in 71% of patients and IV in 29% of patients. Portal vein thrombus (10%), lymphovascular invasion (4%), and perineural invasion (1%) were detected in a minority of patients. The median CA 19–9 level was 54 (interquartile range (IQR): 22–197) units/milliliter.

Systemic therapy prior to RT, most often induction chemotherapy with a gemcitabine/platinum regimen ± nab-paclitaxel, was delivered to 81% of patients. Systemic therapy was given to 58% of patients at some point in their disease course following RT. RT technique was photon-based in 73% and proton-based in 27%, at the discretion of the treating radiation oncologist. The median gross tumor volume (GTV) was 168 (IQR 70–350) cm^3^, and the median planning target volume was 350 (IQR 157–662) cm^3^. The median RT dose was 67.5 (range: 58–100) Gy delivered in a median 15 (range: 10–28) fractions for a median BED_10_ of 98 (range: 81–144) Gy.

### 3.2. Mutational Profiling Results

Of the 156 patients, 114 (73%) had mutational profiling information available. The most commonly found mutations were *IDH1* in twenty-eight (25%) patients, *TP53* in twenty-five (22%), *ARID1A* in twenty-two (19%), *FGFR2* in fifteen (13%), *BAP1* and *IDH2* each in fourteen (12%), and *NRAS*, *CDKN2A*, *MLL2*, and *PIK3CA* each in nine (8%). A list of mutations observed in five or more patients is shown in Supplementary Materials Table S1. The most common pairs of mutations (Supplementary Materials Figure S1) observed were *IDH1*-*ARID1A* in nine patients (8%), *IDH1*-*TP53* in six (5%), and *TP53*-*ARID1A* in five (4%).

### 3.3. Disease Control and Survival

**Figure 1:**
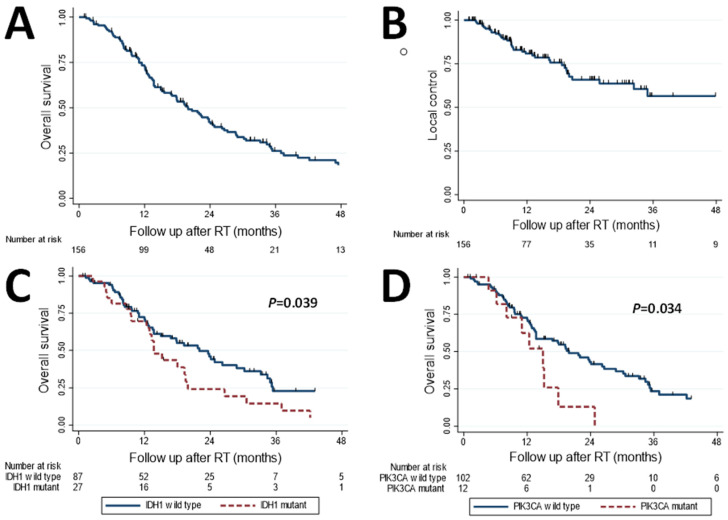
(**A**) Overall survival and (**B**) local control for the entire cohort, and overall survival stratified by (**C**) *IDH1* mutational status and (**D**) *PIK3CA* mutational status.

**Figure 2:**
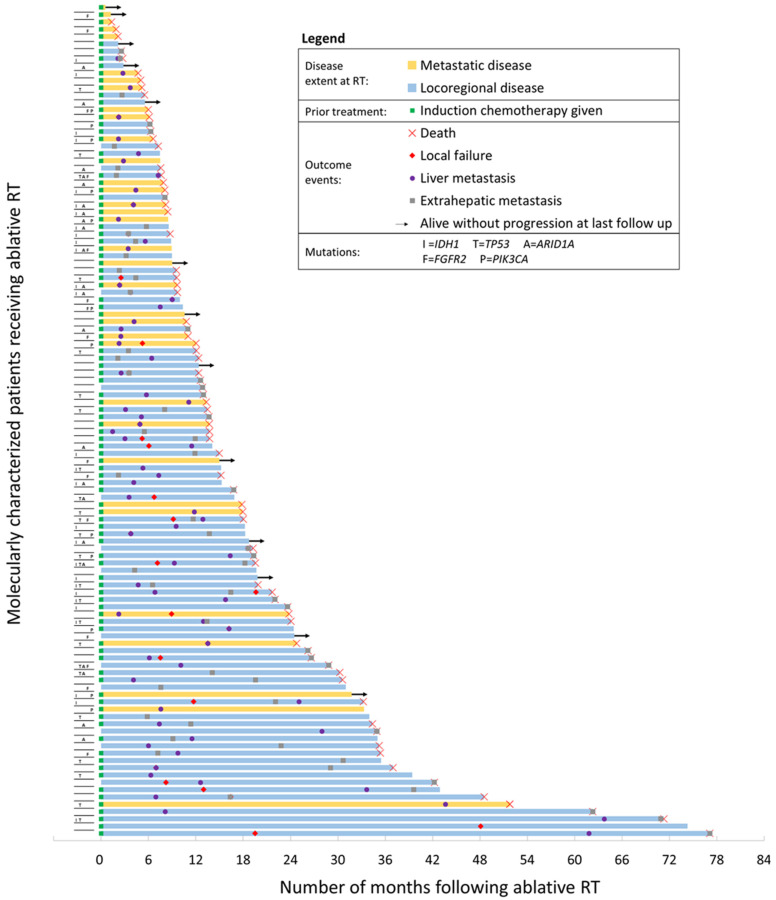
Patient-level outcomes for 114 patients receiving ablative radiotherapy for treatment of intrahepatic cholangiocarcinoma. The latency between radiotherapy and outcome, either last follow-up or death, is represented by the length of each bar shown. Mutation statuses for common mutations are provided.

Estimates of patient outcomes at 1 year following RT, stratified by the four most commonly seen mutations, are shown in Table 2. Outcomes were favorable among all patients, with OS (Figure 1A) and LC (Figure 1B) estimated to be 73% (95% confidence interval (CI): 65–80%) and 81% (CI: 73–87%) at 1 year. Among the mutational subgroups, patients with *FGFR2* mutations had the most favorable OS and LC, estimated to be 92% (CI: 57–99%) and 93% (CI: 59–99%) at 1 year. Conversely, patients with *TP53* mutations had the least favorable OS and LC, estimated to be 58% (CI: 35–76%) and 66% (CI: 41–82%) at 1 year. Patients with *TP53* mutations had the poorest distant disease control of the four most common mutations (*IDH1*, *TP53*, *ARID1A*, and *FGFR2*), with intrahepatic and extrahepatic DMFS estimated to be 10% (CI: 2–26%) and 44% (CI: 19–67%) for these patients. Patient-level outcomes following A-RT are provided in a swimmer plot in Figure 2.

We, therefore, proceeded with univariate Cox proportional hazards of clinical and pathologic factors, the results of which are displayed in Table 3. The attributes associated with an increased risk of death included higher ECOG performance status, larger tumors, metastatic disease at the time of RT, the presence of portal vein thrombus, the presence of satellite lesions, *IDH1* mutation, and *PIK3CA* mutation. The only factor upon univariate analysis associated with a decreased risk of death was a higher dose delivered to 90% of the GTV (D90% GTV). Upon multivariable analysis shown in Table 4, worse performance status (hazard ratio [HR] 1.81; *P* = 0.021), metastatic disease (HR 2.00; *P* = 0.012), higher CA 19-9 levels (HR 1.0001, *P* < 0.001), and mutations in *IDH1* (HR 1.80; *P* = 0.042) and *PIK3CA* (HR 2.29; *P* = 0.034) continued to be associated with an increased risk of death. Additionally, D90% to GTV was associated with a decreased risk of death (HR 0.96; *P* = 0.018). Kaplan–Meier curves of OS among patients, stratified by *IDH1* (Figure 1C) and *PIK3CA* mutations (Figure 1D), are provided.

Factors associated with local recurrence upon univariate analysis (Table 3) included the presence of satellite lesions, lymphovascular invasion, and *TP53* mutation. Upon multivariate analysis (Table 4), mutation in *TP53* (HR 2.41; *P* = 0.041) was found to be significantly associated with local recurrence. Satellitosis (HR 2.63; *P* = 0.054) trended towards significant association with worse local control.

Increasing tumor size, extrahepatic disease, higher CA 19-9 levels, portal vein thrombus, the presence of satellite lesions, and *IDH1*, *TP53*, and *NRAS* mutations were all significantly associated with an increased likelihood of intrahepatic distant metastasis. Higher T-stage, higher CA 19-9 levels, portal vein thrombus, and *PIK3CA* mutation were significantly associated with an increased likelihood of extrahepatic distant metastasis, whereas D90% to GTV was significantly associated with a decreased likelihood.

Among the twenty-eight patients with *IDH1* mutations, eight (29%) received an *IDH1* inhibitor at any point in their disease course. Among the fifteen patients with *FGFR2* fusions/mutations, eight (53%) received an *FGFR* inhibitor at any point in their disease course. *IDH1* and *FGFR2* inhibitors were most commonly used as salvage therapy on a clinical trial following relapse. There were no significant differences in the OS between patients who received *IDH1* or *FGFR* inhibitors versus those who did not (Supplementary Materials Figure S2).

## 4. Discussion

In the current study of 156 patients with unresectable intrahepatic cholangiocarcinoma, we demonstrate favorable LC and OS outcomes following A-RT, with 1-year rates of 81% and 73%, respectively. We characterized the mutational landscape of 114 patients, showing that *IDH1*, *TP53*, *ARID1A*, and *FGFR2* mutations were most commonly found on next generation sequencing and that common co-occurring mutations included *IDH1*/*ARID1A*, *IDH1*/*TP53*, and *ARID1A*/*TP53*. Finally, we provide initial benchmark data showing differential outcomes associated with mutation status, including possible associations of *IDH1* and *PIK3CA* mutations with poorer OS and association of *TP53* mutations with poorer LC.

### 4.1. Hypofractionated RT for ICC

The present analysis provides a contemporary update of our experience with unresectable ICC patients treated with A-RT. In a prior report, patients treated with BED_10_ ≥ 80.5 Gy were shown to exhibit more durable LC and longer OS than those who received lower doses, owing to reductions in rates of TRLF [13]. Since its publication, several other series have added to our understanding of the management of unresectable/locally recurrent ICC and offer findings concordant with those reported in the present study. Though randomized trials are lacking, some prospective data are provided by a phase II trial of both hepatocellular carcinoma and ICC patients receiving hypofractionated proton beam therapy. Two-year LC, progression-free survival (PFS), and OS were noted to be 94%, 26%, and 47%, respectively [23]. A retrospective analysis of 66 patients treated at Massachusetts General Hospital with hypofractionated RT to a median BED_10_ of 80.5 (range: 47–98) Gy showed 2-year LC and OS rates of 84% and 58%, respectively. The authors of the study found that female sex and prior chemotherapy were independently associated with longer OS, whereas prior surgery and macrovascular invasion were independently associated with a higher risk of local failure. Importantly, only 8% of the patients experienced in-field recurrence, corroborating the durable effect of A-RT on local control [24].

A National Cancer Database (NCDB) study of 2,842 unresectable ICC patients treated from 2004 to 2013 showed that patients treated with chemoradiation had longer OS than those treated with chemotherapy alone (median 13.6 versus 10.5 months; *P* < 0.001). However, this study did not analyze RT dose or include information regarding other important disease-related endpoints, including local or distant disease control. Another NCDB analysis showed favorable outcomes with stereotactic body radiotherapy (SBRT), most commonly 45 Gy in five fractions, when compared with transarterial radioembolization (TARE) or chemoradiation; however, no comparison was made specifically to those patients getting chemoradiation with higher BED [4].

Currently ongoing is the prospective, randomized ABC-07 trial, which is comparing cisplatin and gemcitabine with or without SBRT in patients with intrahepatic or extrahepatic cholangiocarcinoma. In addition to these studies examining radiotherapy, several studies on trans-arterial chemoembolization (TACE), hepatic arterial infusion (HAI), TARE, and radiofrequency ablation (RFA) have shown mixed findings in the treatment of unresectable intrahepatic ICC and remain active areas of investigation [3]. Retrospective comparisons between RT and other modalities have been inconclusive given the wide heterogeneity of patients treated across modalities [25].

### 4.2. Mutation Prevalence in ICC

Molecular profiling efforts over the last decade have revealed substantial mutational heterogeneity across ICC tumors, and an evolving understanding of the prevalence of individual mutations is being developed. The largest of these, reported in abstract form, is a study of 3634 patients with cholangiocarcinomas (number of ICC unspecified), which showed the most common mutations to be *TP53* (31%), *CDK2NA* (29%), *KRAS* (20%), and *ARID1A* (17%) [26]. Similarly, a multi-institutional study of 412 ICC patients revealed the most common genetic aberrations to be observed in *TP53* (27%), *CDK2NA/B* (27%), *KRAS* (22%), *ARID1A* (18%), and *IDH1* (16%) [27]. Another analysis of 760 gallbladder cancers (number of ICC unspecified) showed a high prevalence of mutations in DNA repair genes and found that 87% of the patients had at least one actionable genetic alteration, with 14% of the patients expressing a mutation of a direct DNA repair gene and 63% of the patients expressing that of a caretaker gene [28]. Another study of 260 Japanese patients with biliary tract cancers (145 ICC) corroborated the importance of driver *FGFR2* fusion genes [29]. The most common mutations in the present cohort were *IDH1* (25%), *TP53* (22%), *ARID1A* (19%), and *FGFR2* (13%), estimates that appear to be comparable to the existing literature. It remains unclear if select genes are preferentially expressed in unresectable/locally advanced cases. Such correlations may suggest a marker of more aggressive disease, and larger analyses will be needed to investigate this further.

### 4.3. Significance and Co-Occurrence of Mutations

Alterations in *IDH1/2* have been well described across several disease sites, including ICC, central nervous system tumors, chondrosarcomas, and acute non-lymphocytic leukemias. *IDH1* encodes the NADP (+)-dependent metabolic enzyme isocitrate dehydrogenase, which is involved in the citric acid cycle [30]. Among biliary tract cancers, however, several studies have shown that *IDH1* mutations nearly exclusively occur in ICC [27,31,32,33]. A systematic review pooled analysis of 4214 patients reported a prevalence of 13%, with a higher prevalence in centers in the United States compared to Asian centers (18% versus 9%). Of the 46 publications investigated in this review, eight investigated the possible prognostic significance of mutated *IDH1*; none of these reported statistically significant associations between *IDH1* mutations and OS, PFS, or time to progression [33]. In view of the existing literature, it is notable that the patients with *IDH1* mutations in the present study showed an association with shorter OS, which persisted even when adjusted for other factors in a multivariable analysis. Eleven publications in the systematic review reported the prevalence of co-mutations with *IDH1*, with the most frequently reported being *ARID1A* (22%), *BAP1* (16%), and *PBRM1* (13%) [33]. Our results similarly showed that *ARID1A* (32%) and *BAP1* (11%) mutations frequently co-occurred with *IDH1* mutations. 

The *FGFR* growth factor pathway has similarly been implicated in the pathogenesis of a variety of cancers, including gastric, breast, prostate, and bile duct cancers [34]. Deregulation of *FGFR* signaling, particularly through gene fusions, has been shown to play an important role in tumor progression [35]. Among biliary tract cancers, studies have shown that *FGFR2* gene translocations occur nearly exclusively in the ICC and generally occur in younger patients with a more indolent disease course [27,31,32]. Studies have shown that ICC patients with *FGFR2* fusions have longer survival; one such study of 273 ICC patients (83 with *FGFR* genetic alteration) showed a median OS of 37 versus 20 months for patients with and without *FGFR2* mutation/fusion, a difference that persisted even when excluding patients who received *FGFR*-targeted therapies. We did not find an association between *FGFR2* mutation/fusion and outcomes. The most common mutations coexisting with *FGFR* mutations in this study were *BAP1* (22%) and *CDK2NA/B* (19%) [36]. Our results showed that *FGFR2* mutations most commonly co-occurred with *BRCA2* (27%), *NTRK1* (27%), and *BAP1* (20%).

More commonly seen in gallbladder carcinoma, mutations in *PIK3CA* were present in 8% of the patients in the present study and are estimated to affect approximately 3–9% of all patients with ICC. Compared with *IDH1* and *FGFR2*, more limited information is available regarding the prognostic implications of mutations in the oncogene *PIK3CA* in ICC [3]. As it is an actionable target seen in a variety of tumor types, further investigation into its role in the ICC pathogenesis, prognosis, and treatment combinations with A-RT is warranted [37,38]. Lastly, mutations in *TP53* are well-known drivers of disease development and markers of poor prognosis in ICC [39,40].

### 4.4. Use of Novel Systemic Therapies

*IDH1* inhibitors, most notably ivodesinib, as shown in the ClarIDHy trial, have shown promise in improving survival in patients with cholangiocarcinoma [14]. Similarly, *FGFR2* inhibitors, most notably pemigatinib, as shown in the FIGHT-202 trial, have shown encouraging results in patients with previously treated unresectable ICC [15]. The comparisons in the present study of patients treated with and without *IDH1* and *FGFR2* inhibitors were unrevealing (Supplementary Materials Figure S2), likely due to small subgroup sizes and the use of these therapies in the relapse setting, often on a clinical trial. Patients with microsatellite instability-high (MSI-H) or *NTRK* fusions have benefitted from immune checkpoint inhibitors or *TRK* inhibitors in tumor-agnostic basket trials, and subsets of patients received these treatments in the present study [41,42,43]. Nevertheless, the role and timing of these and other targeted therapies in combination with A-RT has yet to be rigorously investigated.

### 4.5. Limitations

This study has several limitations. While all the patients received A-RT, the patient sample is heterogeneous with regard to the disease extent, high-risk disease factors, such as satellitosis, PVT, PNI, and LVI, and therapies received following A-RT. Given the retrospective nature of this study, there is probable selection bias for those patients who would tolerate and benefit most from A-RT, which may have led to more favorable survival than would have been seen for an unselected population. The sample is heterogeneous with regard to mutational profiling, as well, in part due to the long study period (2008–2020), over which the next-generation sequencing methods evolved considerably. The subgroup sizes for molecular mutations were small, which limited our ability to make robust conclusions about their prognostic value. The comparisons for pairs of mutations were similarly limited by small subgroup sizes. While we made attempts to control for clinical and pathologic factors through the use of multivariable models, it is nevertheless challenging to draw definitive conclusions about the independent prognostic value of an individual mutation. Additionally, we are unable to offer a mechanistic explanation relating gene mutations to the differential outcomes seen in the context of ablative radiotherapy. Lastly, these data may not be generalizable to specific populations with different disease etiologies and pathogeneses, such as ICC associated with liver flukes, viral hepatitis, autoimmune disease, or metabolic syndromes.

## 5. Conclusions

Advancements in the treatment of ICC over the last decade have increasingly involved the use of local therapies as well as stratification and treatment according to mutational status. However, this is the first analysis that seeks to combine these parallel efforts. In the present study, we stratified patients with unresectable ICC by genetic alterations to provide benchmarks for future analysis and comparison. Compared to the historical outcomes for ICC patients, favorable outcomes were observed across molecular profiles in the present analysis. We also demonstrated that *IDH1* and *PIK3CA* mutations may be associated with poorer survival, and *TP53* mutations may be associated with poorer local control for patients with ICC receiving A-RT. Further investigation into the prognostic value and therapeutic implications of individual mutations and combinations thereof is warranted.

## Figures and Tables

**Table 1:** Baseline patient, disease, and treatment characteristics.

Attribute	Value
Female sex	54%
Median age at start of radiotherapy (range)	66 (31–89)
Median number of tumors (range)	1 (1–5)
Satellitosis	51%
Median dominant tumor size (range, cm)	7.3 (2.2–18.2)
ECOG Performance Status	
0	42 (27%)
1	103 (66%)
2	11 (7%)
AJCC 8th Edition Stage *	
I	12%
II	22%
III	38%
IV	29%
Portal vein thrombus	10%
Lymphovascular invasion (*n* = 143)	5 (4%)
Perineural invasion (*n* = 143)	2 (1%)
Median CA 19-9 level (IQR, units/mL)	54 (22–197)
Systemic therapy	
Before RT	81%
During RT	63%
After RT	58%
RT technique	
Photon	73%
Proton	27%
Median gross tumor volume (cm^3^, IQR)	168 (70–350)
Planning target volume (cm^3^, IQR)	350 (157–662)
Median RT dose (range, Gy)	67.5 (58–100)
Median RT fractions (range)	15 (10–28)
Median RT BED_10_ (range, Gy)	98 (81–144)

ECOG = Eastern Cooperative Oncology Group, AJCC = American Joint Commission on Cancer, IQR = interquartile range, RT = radiotherapy, BED = biologically effective dose. * Numbers add to >100% due to rounding.

**Table 2:** Time-to-event outcomes of patients stratified by the most commonly mutated genes.

	Outcomes (95% CI) at 1 Year Following RT			
Mutation Status	OS	LC	Intrahepatic DMFS	Extrahepatic DMFS
*IDH1* mutant (*n* = 28)	70% (48–84%)	64% (40–80%)	16% (5–34%)	50% (27–69%)
*TP53* mutant (*n* = 25)	58% (35–76%)	66% (41–82%)	10% (2–26%)	44% (19–67%)
*ARID1A* mutant (*n* = 22)	77% (53–90%)	74% (48–88%)	32% (14–51%)	61% (33–80%)
*FGFR2* mutant/fusion (*n* = 15)	92% (57–99%)	93% (59–99%)	15% (2–37%)	50% (18–75%)
All patients (*n* = 156)	73% (65–80%)	81% (73–87%)	34% (26–42%)	60% (50–68%)

RT = radiotherapy, OS = overall survival, LC = local control, DMFS = distant metastasis-free survival.

**Table 3:** Univariate Cox analysis of factors associated with time-to-event outcomes following initiation of ablative radiotherapy.

Attribute	OS		LC		Intrahepatic DMFS		Extrahepatic DMFS	
	HR	*P*-Value	HR	*P*-Value	HR	*P*-Value	HR	*P*-Value
Female sex	0.75	0.146	1.61	0.173	0.87	0.437	0.86	0.493
Performance status	1.47	0.021 *	1.08	0.774	1.19	0.224	1.27	0.168
Tumor size	1.06	0.043 *	0.99	0.861	1.08	0.003 *	1.03	0.444
T-stage	1.12	0.300	1.32	0.110	1.17	0.118	1.29	0.031 *
N-stage	1.37	0.102	0.92	0.800	1.29	0.144	1.21	0.375
M1 disease at RT	2.15	<0.001 *	1.65	0.183	1.80	0.003 *	-	-
CA 19-9	1.0001	<0.001 *	1.0001	0.517	1.0001	0.001 *	1.0004	0.005 *
PVT	2.30	0.008 *	1.15	0.821	1.93	0.018 *	2.46	0.041 *
Satellitosis	1.63	0.013 *	2.57	0.006 *	1.61	0.008 *	1.50	0.060
Lymphovascular invasion	1.31	0.602	3.44	0.044 *	1.08	0.886	1.13	0.815
Proton RT technique	0.76	0.201	0.45	0.060	0.92	0.661	0.88	0.556
D90% to GTV	0.97	0.005 *	1.00	0.996	0.98	0.060	0.96	0.006 *
*IDH1* mutation	1.68	0.041 *	2.07	0.079	1.71	0.028 *	1.68	0.063
*TP53* mutation	1.53	0.136	2.35	0.035 *	1.72	0.031 *	1.31	0.422
*ARID1A* mutation	1.53	0.109	1.46	0.386	1.42	0.153	1.27	0.426
*FGFR2* mutation/fusion	0.60	0.154	0.32	0.118	1.19	0.544	0.87	0.719
*BAP1* mutation	0.62	0.232	1.04	0.948	1.10	0.748	0.69	0.367
*IDH2* mutation	1.00	0.990	1.73	0.235	0.74	0.348	1.12	0.777
*NRAS* mutation	0.73	0.596	2.87	0.097	3.23	0.004 *	0.64	0.536
*CDKN2A* mutation	1.11	0.783	1.90	0.235	1.25	0.529	0.86	0.730
*MLL2* mutation	0.99	0.967	1.81	0.229	1.02	0.948	0.86	0.743
*PIK3CA* mutation	2.13	0.039 *	1.50	0.514	1.49	0.219	2.56	0.035 *
No mutations	0.72	0.370	0.62	0.429	0.65	0.168	0.474	0.086

HR = hazard ratio, M1 = metastatic, D90% GTV = dose delivered to 90% of gross tumor volume, PVT = portal vein thrombus, OS = overall survival, LC = local control, DMFS = distant metastasis-free survival. * Significant at *P* < 0.05.

**Table 4:** Multivariable Cox analysis of factors associated with overall survival and local control following initiation of ablative radiotherapy. Cells corresponding to variables that did not meet criteria for inclusion in the multivariable model are left blank.

Attribute	OS		LC	
	HR	*P*-Value	HR	*P*-Value
Female sex				
Performance status	1.81	0.021 *		
Tumor size	1.02	0.578		
T-stage				
N-stage				
M1 disease at RT	2.00	0.012 *		
CA 19-9	1.0001	<0.001 *		
PVT	2.11	0.069		
Satellitosis	1.56	0.110	2.63	0.054
Lymphovascular invasion			3.96	0.091
Proton RT technique			0.63	0.374
D90% to GTV	0.96	0.018 *		
*IDH1* mutation	1.80	0.042 *	1.28	0.601
*TP53* mutation			2.41	0.041 *
*ARID1A* mutation				
*FGFR2* mutation/fusion				
*NRAS* mutation			2.21	0.232
*PIK3CA* mutation	2.29	0.034 *		

HR = hazard ratio, M1 = metastatic, D90% GTV = dose delivered to 90% of gross tumor volume, PVT = portal vein thrombus, OS = overall survival, LC = local control. * Significant at *P* < 0.05.

## Data Availability

The data that support the findings of this study are available from the corresponding author, E.J.K., upon reasonable request within 1 year of publication.

## Supplementary Materials

The following are available online at https://www.mdpi.com/article/10.3390/jpm11121270/s1, Figure S1: Co-occurrence of mutated genes, Figure S2: Overall survival of patients with (a) *IDH1* mutations and (b) *FGFR2* mutations stratified by receipt of molecularly targeted therapy, Table S1: List of mutations with frequencies of 5 or greater.
