# Supplementary material for: Benchmarking Outcomes after Ablative Radiotherapy for Molecularly Characterized Intrahepatic Cholangiocarcinoma
Source: J Pers Med. 2021 Dec 1;11(12):1270. doi: 10.3390/jpm11121270 (PMC8703854; doi:10.3390/jpm11121270)
Supplement: Supplementary file 1 [file jpm-11-01270-s001.zip › jpm-1471939-supplementary.pdf]

Figure S1. Co-occurrence of mutated genes.

	IDH1	IDH2	TP53	ARID1A	FGFR2	BAP1	NRAS	MLL	PIK3CA	BRCA2	KRAS	NTRK1	ERBB2	ATM
IDH1		2	6	9	1	3	2	4	3	2	3	2	1	1
IDH2			3	2	1	1	1	2	3	2	0	0	0	0
TP53				5	3	3	2	1	2	2	1	1	2	3
ARID1A					3	3	2	4	1	0	2	1	2	0
FGFR2						3	1	0	2	4	1	4	1	2
BAP1							1	3	1	0	1	1	1	0
NRAS								1	1	1	1	1	0	0
MLL									3	0	0	2	1	0
PIK3CA										2	0	0	0	1
BRCA2											0	2	0	2
KRAS												0	1	0
NTRK1													1	1
ERBB2														1
ATM														

Figure S2. Overall survival of patients with (A) *IDH1* mutations and (B) *FGFR2* mutations stratified by receipt of molecularly targeted therapy.

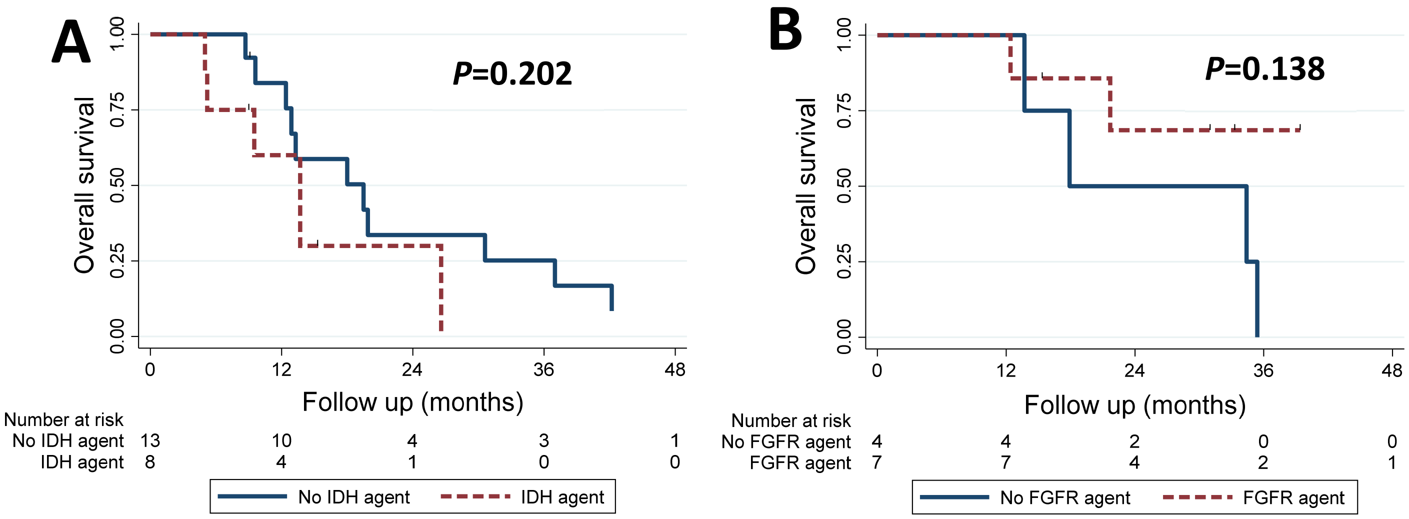

Table S1. List of mutations with frequencies of 5 or greater.

Gene altered	Frequency (%)
IDH1	28 (25%)
TP53	25 (22%)
ARID1A	22 (19%)
FGFR2	15 (13%)
None	15 (13%)
BAP1	14 (12%)
IDH2	14 (12%)
NRAS	9 (8%)
CDKN2A	9 (8%)
MLL2	9 (8%)
PIK3CA	9 (8%)
BRCA2	8 (7%)
NTRK1	7 (6%)
ATM	7 (6%)
KRAS	7 (6%)
ERBB2	6 (5%)
PBRM1	6 (5%)
EP300	6 (5%)
KIT	5 (4%)
MET	5 (4%)
APC	5 (4%)
LRP1B	5 (4%)
NF1	5 (4%)
NOTCH3	5 (4%)
